# Effectiveness of a Plan-Do-Study-Act cycle-based quality control circle in enhancing the research capacity of operating room nurses a pre-post intervention study

**DOI:** 10.1186/s12912-026-04566-3

**Published:** 2026-03-25

**Authors:** Xiaofen Yu, Xinyu Wang, Yanlin Yang, Wenwen Hou, Zheng Wang

**Affiliations:** 1 Cancer Center, Department of the Operating Room, Zhejiang Provincial People’s Hospital (Affiliated People’s Hospital of Hangzhou Medical College), Hangzhou, Zhejiang Province China; 2 Xiasha Campus of Hangzhou First People’s Hospital (Hangzhou Rehabilitation Hospital), Hangzhou, Zhejiang Province China; 3 Cancer Center, Zhejiang Provincial People’s Hospital (Affiliated People’s Hospital of Hangzhou Medical College), Hangzhou, Zhejiang Province China

**Keywords:** PDSA cycle, Quality control circle, Operating room nurses, Research capacity, Nursing education

## Abstract

**Background:**

Enhancing the research capacity of operating room nurses is essential for advancing evidence-based practice, yet effective and systematic training strategies are lacking. This study evaluated the efficacy of a Quality Control Circle (QCC) intervention structured around an enhanced Plan-Do-Study-Act (PDSA) cycle in improving nurses’ research competency and scholarly output.

**Methods:**

A pre-post intervention study was conducted in 2022, A convenience sampling approach was adopted in this study, with no control group due to the department-wide quality improvement nature of the intervention. A 9-member QCC team designed and implemented a structured training program for 103 operating room nurses, incorporating research methodology workshops and PDSA-guided mentorship. The primary outcome was the change in self-assessed research competency scores (scale: 0-120). Secondary outcomes included research outputs (granted projects, publications in core and first-tier journals) tracked through 2024. Data were analyzed using paired t-tests or Wilcoxon signed-rank tests as appropriate.

**Ethical approval:**

Granted by the Ethics Committee of Zhejiang Provincial People’s Hospital (Approval No: KT2024025). Written informed consent was obtained from all participants.

**Results:**

Participants’ median self-assessed research competency score increased significantly from 58.0 (IQR: 49.0–67.0) pre-intervention to 111.0 (IQR: 79.0–120.0) post-intervention (*P* < 0.001). Research outputs demonstrated marked improvement: funded projects increased from 2 to 10 (*P* = 0.047), core journal publications from 4 to 14 (*P* = 0.041), and first-tier journal publications from 0 to 6 (*P* = 0.031). QCC members also reported significant gains in intangible skills such as problem-solving and teamwork (all *P* < 0.05).

**Conclusions:**

A QCC intervention underpinned by an enhanced PDSA cycle is associated with significant improvements in the research capacity, productivity, and associated competencies of operating room nurses. This model offers a feasible framework for cultivating a sustainable research culture in surgical nursing settings.

**Clinical trial number:**

Not applicable.

**Supplementary Information:**

The online version contains supplementary material available at 10.1186/s12912-026-04566-3.

## Background

Nursing research forms the foundation for disciplinary advancement and evidence-based practice. In China, however, the research capacity among clinical nurses remains underdeveloped [1, 2]. National surveys indicate that only 7.9% of nurses have engaged in research projects, and a mere 5.4% have published academic papers [1].Operating room nurses confront exacerbated barriers due to their unique work environment—characterized by high intensity, demand for multidisciplinary collaboration, and a traditional emphasis on technical proficiency over scholarly inquiry [3–5]. For instance, less than 10% of operating room nurses report receiving systematic research training, leaving numerous clinical challenges, such as optimizing perioperative processes, unaddressed by empirical investigation [4]. This gap not only impedes nurses’ professional growth but also limits innovation within specialized surgical nursing practice. Quality Control Circles (QCC) and the Plan-Do-Study-Act (PDSA) cycle are established quality improvement tools in healthcare, frequently employed to resolve clinical issues and enhance service quality [6, 7]. Conventional applications of the PDSA cycle, however, often treat the “Study” phase superficially, focusing on outcome evaluation rather than the deliberate cultivation of research-oriented thinking and skills [8]. While isolated studies have employed QCC or PDSA frameworks to bolster research capacity, robust evidence on integrating QCC with a comprehensively enhanced PDSA cycle to systematically develop research competence among operating room nurses is scarce [9, 10]. Therefore, this study aimed to evaluate the effectiveness of an enhanced PDSA-based QCC intervention in improving operating room nurses’ research capability and scholarly output, providing a replicable model for fostering research engagement in similar clinical environments.

## Methods

### Study design and participants

Operating room nurses face significant barriers to research capacity development, including high clinical workloads and limited access to systematic training. This department-level quality improvement study was designed to evaluate the effectiveness of a Plan-Do-Study-Act (PDSA) cycle-based intervention in enhancing nurses’ research competency, with all eligible participants enrolled to ensure the generalizability of results to real clinical practice. This pre-post intervention study enrolled all eligible operating room nurses (*n* = 103) from a tertiary hospital in January 2022. Inclusion criteria comprised: (1) active clinical practice in the operating room, and (2) provision of written informed consent. Nurses absent from their position for ≥ 2 months during the study were excluded. The cohort consisted of 14 males and 89 females, aged 20–53 years, all holding a bachelor’s degree. Professional titles included Nurse-in-Charge (*n* = 52), Senior Nurse (*n* = 31), and Nurse (*n* = 20).

Ethical Considerations: The study was approved by the Institutional Review Board of Zhejiang Provincial People’s Hospital (Approval No: KT2024025). Participation was voluntary. All participants received comprehensive oral and written information regarding the study’s purpose, procedures, their right to withdraw without penalty, and guarantees of data anonymity and confidentiality, in accordance with the Declaration of Helsinki.

Sample Size: As a departmental quality improvement initiative aiming to involve all eligible staff, a convenience sampling approach was used without a priori sample size calculation. This is common in such contexts where the primary objective is to assess feasibility and effect within a specific unit. Consequently, statistical power, particularly for secondary outcomes, may be limited, and findings should be interpreted as preliminary evidence from a single-center setting.

### Intervention

#### Overview and development

Following a baseline assessment that pinpointed core competency gaps, a quantitative improvement target was established using a standard quality improvement formula, informed by team self-assessment. A structured intervention was then designed and executed by a spontaneously formed 9-member QCC team. This team included the Nursing Director (facilitator), the Operating Room Head Nurse (leader), five clinical nursing experts, and two research nurses with master’s degrees, ensuring a blend of managerial, clinical, and academic expertise. The program was structured around an enhanced PDSA cycle framework, Detailed intraoperative parameters are provided in Table 1.

**Table 1:** PDSA Cycle-based structured training program for operating room nurses

PDSA Phase	Core Objectives / Themes	Key Activities / Methods	Duration / Frequency	Facilitator(s)	Expected Outputs / Evaluation Methods
Plan (P) (Months 1–3)	Problem diagnosis and program planning	1. Baseline assessment using the Nursing Staff Research Capacity Self-Assessment Scale [11].	3 months	QCC members, Nursing Department	Clearly defined improvement priorities, quantified target goals, a structured training program.
		2. Brainstorming and root cause analysis (e.g., fishbone diagram).			
		3.Development of a structured training plan.			
Do (D) (Months 4–10)	Systematic theoretical and skills training	Module 1: Research Design – Core lectures by academic faculty; seminars on translating clinical questions into research.	7 months (ongoing)	QCC leader, invited lecturers, senior nurses	Nurse participation rates, training feedback, formative learning outcomes (e.g., reflection reports, simulated research protocols).
		Module 2: Scientific Writing & Literature Review – critical appraisals of high-quality papers; simulated research design workshops.			
		Module 3: Research Environment & Support – Improved library/database access; fostering an English-language learning atmosphere.			
Study (S) (Ongoing)	Process monitoring and personalized support	1. One-on-one mentoring for nurses developing projects/papers.	Throughout implementation	Postgraduate nurses, research committee members	Records of personalized mentoring sessions, learning progress reports, periodic self-assessments of competency.
		2. Learning style assessment (using Kolb’s LSI [12]) to adapt teaching methods.			
		3. Progress tracking via a digital collaboration platform (Ding Talk).			
Act (A) (Months 11–12 & beyond)	Standardization and sustainability	1. Establishment of a permanent nursing research committee.	Final 2 months & ongoing	Head research nurse, committee chair	Standardized operating procedures (SOPs), data on sustained research output, updated annual training program.
		2. Standardized procedures for annual scholarly output expectations.			
		3. Annual evaluation and iterative revision of the training program.			

#### Needs assessment

**Fig. 1:**
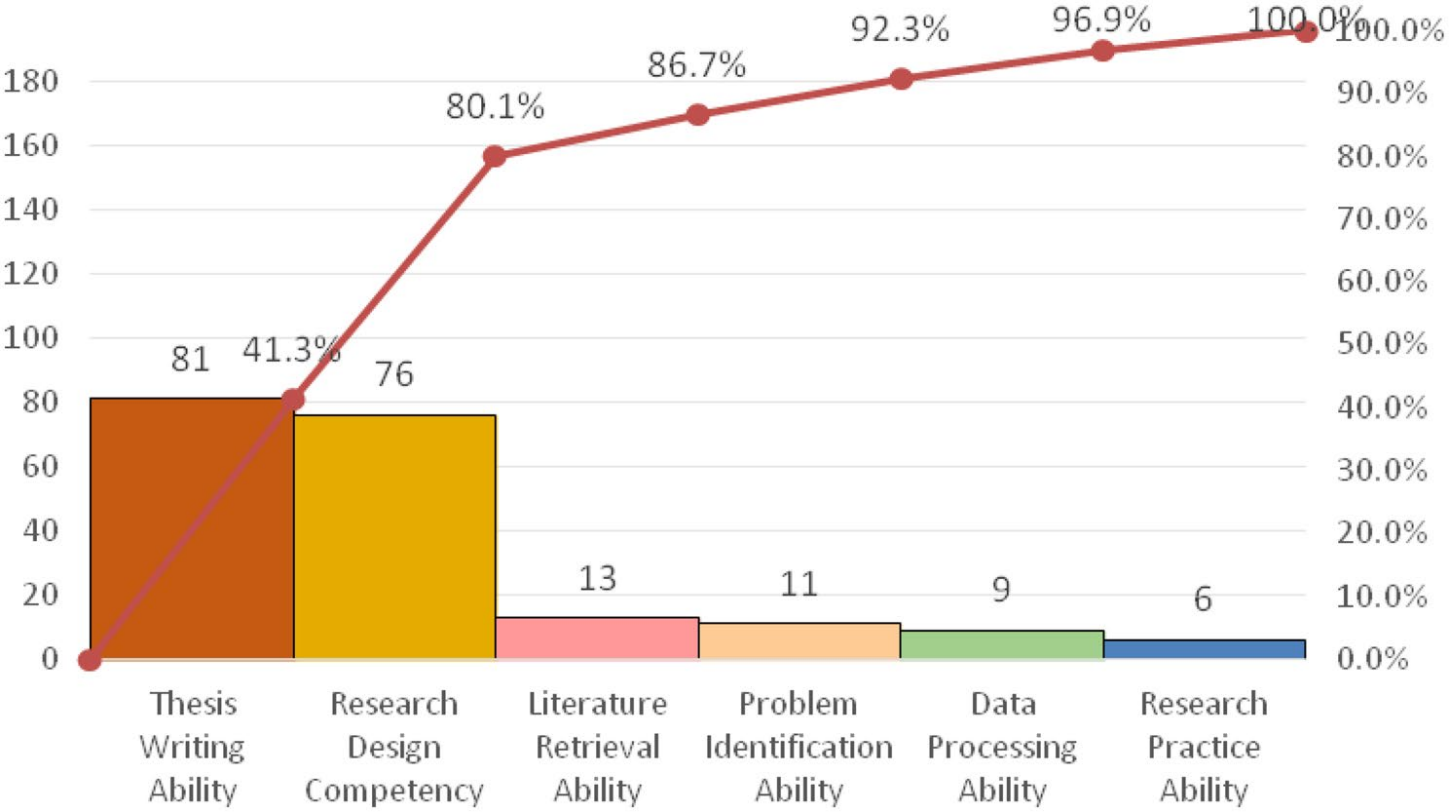
Pareto analysis of research competency deficiencies before the intervention

Root cause analysis, employing a fishbone diagram and a survey of all 103 nurses, identified “insufficient research design competency” and “insufficient scientific writing competency” as the primary deficits, Five key contributing barriers were isolated: lack of systematic training, limited training methods, insufficient practical opportunity, poor literature access, and weak foreign language skills, See Tables 2 and 3; Fig. 1.

**Table 2:** Distribution of self-assessed research competency levels among operating room nurses at baseline (*n* = 103)

Dimension	Competent	Moderately Competent	Insufficiently Competent	Total
Thesis Writing Ability	3(2.9)	19(18.4)	81(78.6)	103
Research Design Competency	2(1.9)	25(24.3)	76(73.8)	103
Literature Retrieval Ability	10(9.7)	80(77.7)	13(12.6)	103
Problem Identification Ability	5(4.9)	87(84.5)	11(10.7)	103
Data Processing Ability	5(4.9)	89(86.4)	9(8.7)	103
Research Practice Ability	3(2.9)	94(91.3)	6(5.8)	103

**Table 3:** Distribution and cumulative proportion of identified research competency deficiencies before the intervention (*n* = 196)

Deficient Dimension	Frequency (*n*)	Proportion (%)	Cumulative Proportion (%)
Thesis Writing Ability	81	41.3	41.3
Research Design Competency	76	38.8	80.1
Literature Retrieval Ability	13	6.6	86.7
Problem Identification Ability	11	5.6	92.3
Data Processing Ability	9	4.6	96.9
Research Practice Ability	6	3.1	100.0

**Fig. 2:**
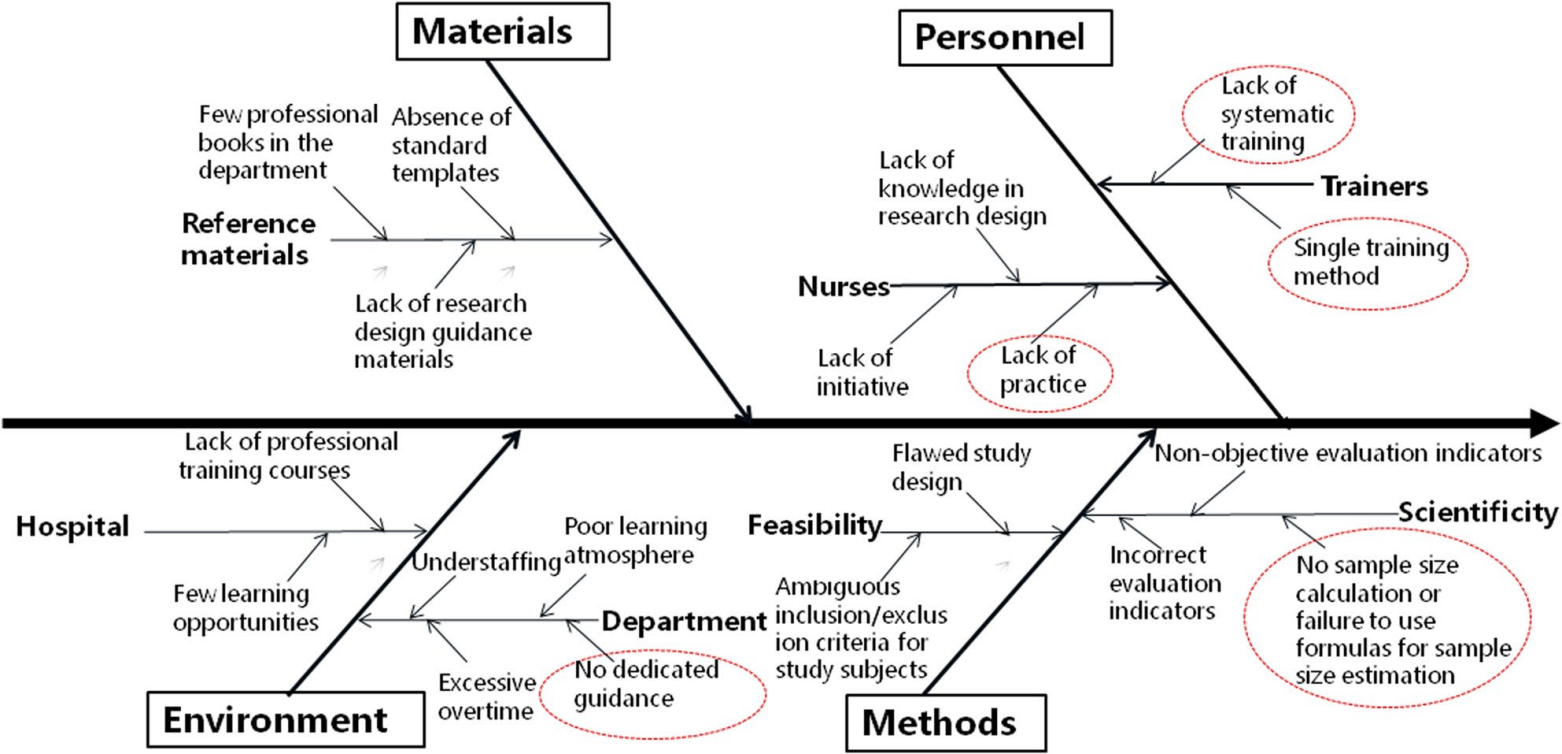
Fishbone diagram for root cause analysis of insufficient research. Design competence (n=103)

**Fig. 3:**
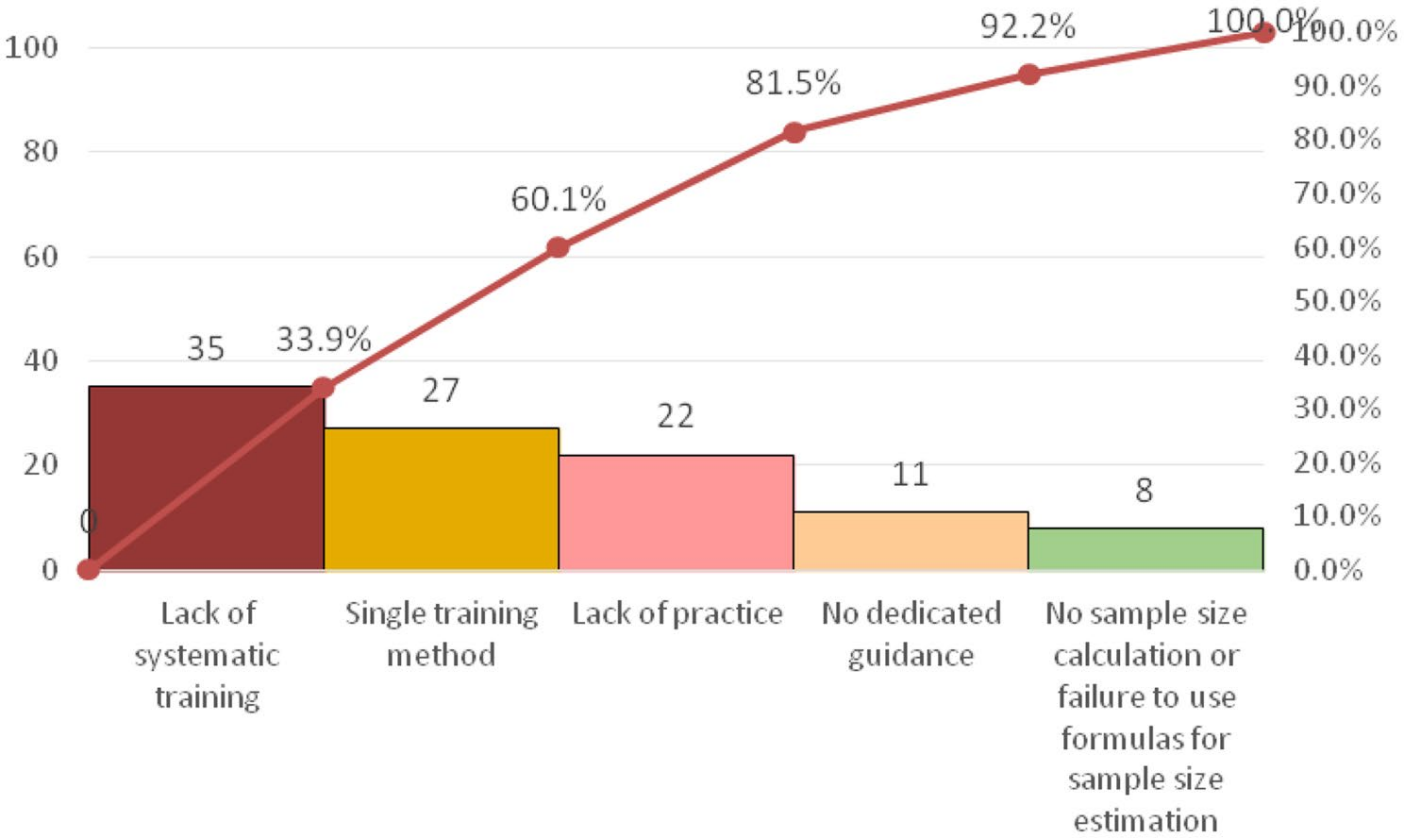
Pareto chart for root cause validation of insufficient research design. Capability (n = 103)

**Fig. 4:**
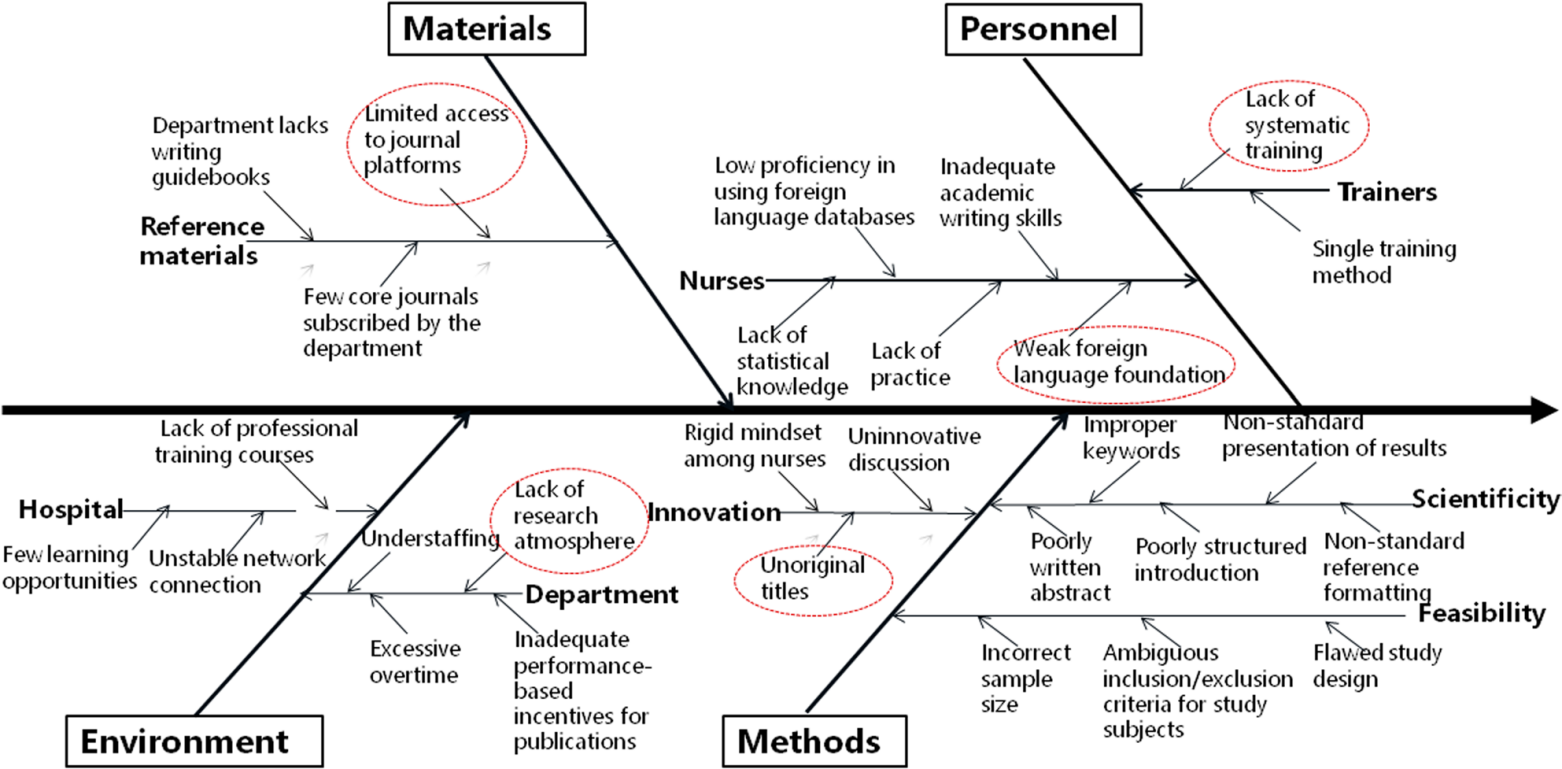
Fishbone diagram for root cause analysis of insufficient thesis writing competence (*n* = 103)

**Fig. 5:**
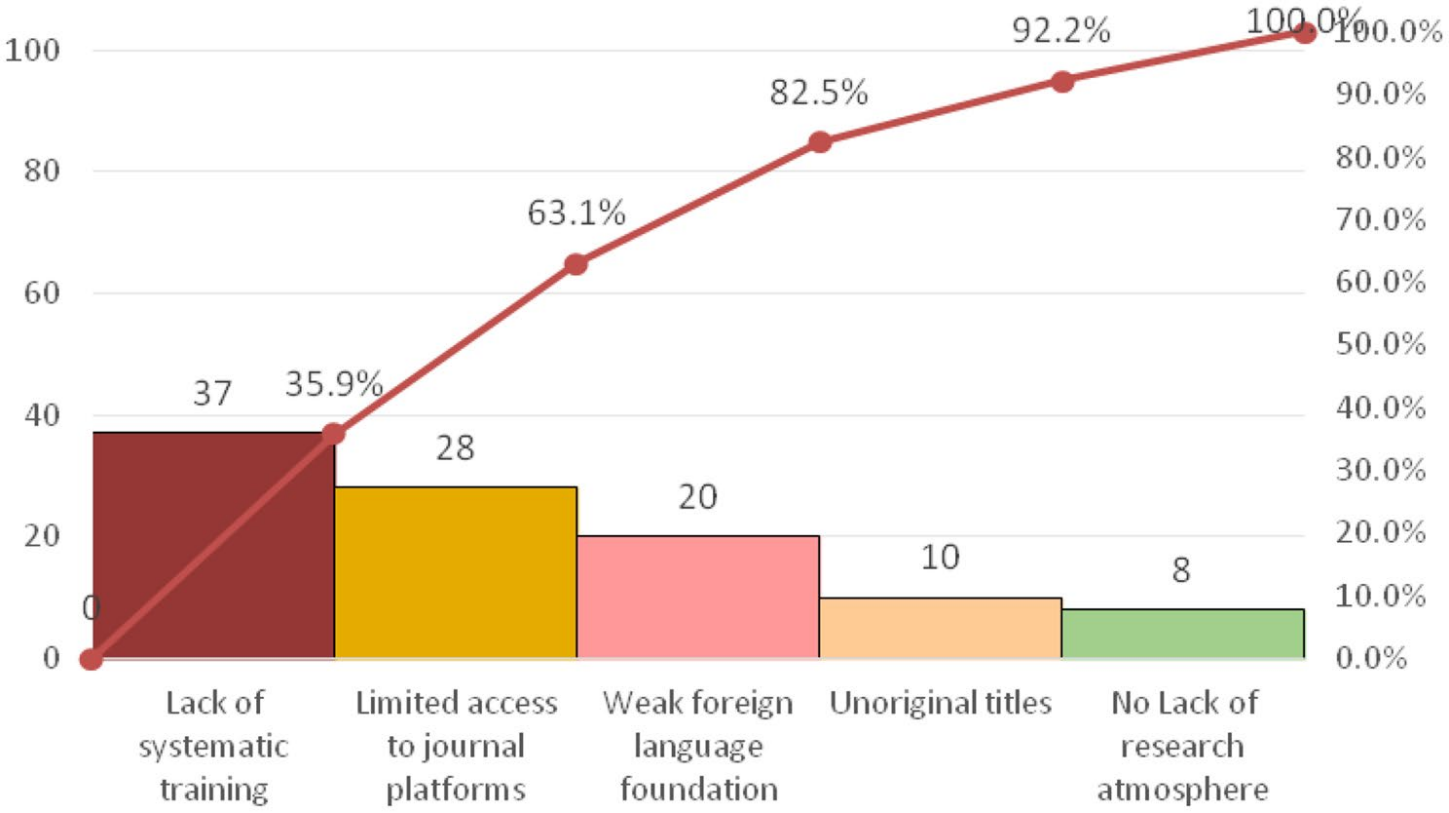
Pareto chart for root cause validation of insufficient manuscript writing ability (*n* = 103)

The root causes and distribution characteristics of insufficient research and writing competence before intervention were visualized in Figs. 2, 3, 4 and 5. Among them, Figs. 2 and 4 show the fishbone diagrams for root cause analysis, while Figs. 3 and 5 present the results of Pareto distribution analysis.

#### Program implementation

The hybrid training program was delivered over 12 months (January–December 2022), coordinated and iteratively reviewed through QCC meetings. Implementation followed the four phases of the PDSA cycle, as detailed in Table 1.

(1) Plan phase (months 1–3): problem diagnosis and program design

This phase focused on comprehensive problem analysis and intervention planning. It commenced with a baseline assessment using the Nursing Staff Research Capacity Self-Assessment Scale [11]. Subsequent root cause analysis via brainstorming and a fishbone diagram led to the evaluation and prioritization of improvement strategies by the QCC team based on feasibility and impact. The outcome was a structured, PDSA-based hybrid training program with quantified targets (Table 1).

(2) Do phase (months 4–10): systematic theoretical and skills training

The specific training activities in this phase were systematically executed based on the pre-defined key issues, root causes, and prioritized countermeasures formulated during the Plan phase. This ensured that the training was tightly aligned with the identified needs and focused on the highest-yield strategies.

A 7-month intensive, diversified, and modular hybrid training program was subsequently delivered. The three core modules were designed to operationalize these countermeasures:

Module 1 (Research Design): Featured core lectures by academic faculty and research experience seminars.

Module 2 (Scientific Writing & Literature Review): Included critical appraisals of high-quality literature and simulated research design workshops.

Module 3 (Research Environment & Support): Focused on improving database access and fostering an English-language learning atmosphere.

All sessions utilized the platform for live streaming and asynchronous access.

(3) Study phase (ongoing, concurrent with do phase): process monitoring and personalized support

This phase emphasized continuous oversight and tailored guidance, including:

One-on-one research mentoring by postgraduate nurses.

Learning style assessment using Kolb’s LSI [12] to inform teaching methods.

Continuous progress tracking and feedback collection via the platform (see Table 4).

(4) Act phase (months 11–12 & beyond): standardization and sustainability

The final phase aimed to institutionalize effective practices by:

Establishing a permanent departmental “Nursing Research Committee.”

Developing standardized procedures for annual research output expectations.

Implementing a mechanism for annual program evaluation and iterative refinement based on outcome data.

**Table 4:** Scoring sheet for the analysis of key factors and the formulation of countermeasures to improve operating room nurses’ research capacity

Problem	Root Cause	Proposed Countermeasure	Score
Insufficient Research Design & Manuscript Writing Ability	Lack of systematic training	1. Develop a “Scientific Research Training Program for Operating Room Nurses”, clarifying training objectives, syllabus, content, plan, methods, and evaluation.	121
		2. QCC members conduct intermittent individual interviews to gather feedback on the training program from both trainers and trainee nurses, and make improvements accordingly.	126
Insufficient Research Design Ability	Singular training method	1. Use the Kolb Learning Style Inventory [12] to understand nurses’ learning styles. Form study groups based on learning styles, appoint group leaders who supervise progress towards training goals.	135
		2. Invite faculty from affiliated universities to deliver lectures based on the training program, using slide presentations on nursing research design, with simultaneous live streaming in the group.	135
		3. Organize seminars where principal investigators of departmental projects and authors of core journal publications share experiences on: identifying clinical problems, translating them into research questions, and developing study protocols and outcome measures.	127
		4. For nurses requiring intensive guidance on ongoing projects/papers, the QCC leader arranges one-on-one mentoring by postgraduate students. Upon completion, both mentors and mentees share their experiences	128
	Lack of practical experience	1. QCC members share 1–2 high-impact papers on perioperative nursing in the study group (themes). Each nurse must critically appraise at least one paper, analyzing the alignment of methods with aims, subject selection, outcome measures, sample size calculation, and evaluation criteria. They submit a reflection report. The best report is selected within groups and presented department-wide with commentary from PhD/MSc holders (can be online).	130
		2. QCC members select 3–5 research titles (quantitative, qualitative, evidence-based nursing) relevant to the specialty. Nurses, without consulting references and within a time limit, independently analyze the research aims, conceptualize methods, and draft a research plan. They then compare their work with the original paper for self-reflection (activity).	109
Insufficient Manuscript Writing Ability	Limited access to paper databases/platforms	1. Improve departmental network stability. Encourage nurses to join groups for literature access created by the hospital library or affiliated universities.	119
		2. Apply for access to the hospital library’s off-campus online platform, enabling nurses to search scientific databases from outside the hospital.	109
		3. Purchase research-related book series and subscribe to core journals for the department.	123
	Weak foreign language foundation	1. Encourage the use of English during morning handovers. Postgraduate nurses are required to hand over in English. To ensure comprehension, they must use supporting slides.	110
		2. Encourage the use of English in departmental tiered training sessions. Invite PhD/MSc holders within the circle to provide occasional commentary and guidance.	109
		3. Encourage nurses to critically read at least 2–3 English papers per month.	116
		4. Encourage nurses to switch their interface language to English to immerse themselves in an English-language environment.	120

Note: Countermeasures were scored by QCC members based on criteria such as feasibility and anticipated effectiveness. The maximum possible score was 150, with higher scores indicating higher priority for implementation

### Measures

#### Primary outcome: self-assessed research capacity

Assessed using the validated Nursing Staff Research Capacity Self-Assessment Scale (revised by Pan et al. [11]). This 30-item instrument covers six dimensions (problem discovery, literature review, research design, research practice, data processing, thesis writing) on a 5-point Likert scale (0="unable” to 4="fully able”), yielding a total score of 0-120 (higher score indicates greater capacity). It demonstrates good reliability (Cronbach’s α = 0.86, test-retest reliability = 0.90) in the target population [13]. Assessments were conducted pre-intervention and post-intervention with 100% valid response rates.

#### Secondary outcome: research outputs

Objective output data were collected for the intervention year (2022) and two subsequent years (2023, 2024), including: number of funded projects (as principal investigator), number and tier of first-author publications (core and first-tier journals per hospital list), authorized patents (as first inventor), and conference presentations/submissions.

#### Process evaluation metrics

Goal Achievement and Improvement Rates: Calculated using standard QCC formulas based on the composite “Scientific Research Capacity Xiankuangzhi” (see Section “Calculation of the composite evaluation metric”).

$$\begin{aligned}&\mathrm{Goal\:Achievement\:Rate}\: \cr & \:= [(\mathrm{Post-score} - \mathrm{Pre-score}) / (\mathrm{Target} - \mathrm{Pre-score})]\cr & \: \times 100 \% \\ \cr & \mathrm{Improvement\:Rate}\: \cr & \:= [(\mathrm{Post-score} - \mathrm{Pre-score}) / \mathrm{Pre-score}]\cr & \: \times 100 \%\end{aligned}$$

##### Intangible Outcomes for QCC Members

Assessed via a 9-item, 5-point Likert self-evaluation scale (1= “Very weak” to 5= “Very strong”; total score 45) developed with expert input, measuring changes in learning motivation, teamwork, research mindset, etc.

### Statistical analysis

Data were analyzed using SPSS 23.0, with α = 0.05.

#### Calculation of the composite evaluation metric

A composite “Scientific Research Capacity Xiankuangzhi” was constructed to integrate subjective self-assessment (40% weight) and objective performance data (60% weight). This mixed-methods approach mitigates social desirability bias and aligns with results-oriented quality improvement principles [14–16]. The weighting was determined through QCC team consensus and aligns with frameworks prioritizing outcome indicators.

Calculation:

##### Subjective competency score:

Dimensional scores from the primary scale were converted to ordinal values (1 = Insufficient, 2 = Moderate, 3 = Competent) based on average item scores, then summed across all participants.

##### Objective performance score:

Research outputs were converted to performance points based on hospital metrics.

*Composite Xiankuangzhi* = (Subjective Score / Max Subjective Score × 0.4) + (Objective Score / Target Objective Score × 0.6).

#### Analysis of intervention effects

Descriptive statistics are presented as frequency (%) and median (IQR). Pre-post comparisons of self-assessment scores (non-normal data) used the Wilcoxon signed-rank test. Annual research output comparisons used the Chi-square or Fisher’s exact test. Intangible outcome scores were compared using the Wilcoxon signed-rank test.

See Supplementary Materials for the detailed operational procedures, scoring criteria and internal management workflow of the Quality Control Circle (QCC) intervention.

## Results

### Goal achievement

Post-intervention, the Goal Achievement Rate was 131.8% and the Improvement Rate was 129.8%.

### Comparison of self-assessed research capacity

Post-intervention total and dimensional scores on the research capacity scale were significantly higher than pre-intervention scores (all *P* < 0.001). The distribution of competency levels improved substantially across all dimensions, See Table 5, and median scores demonstrated marked increases, See Table 6.

**Table 5:** Distribution of self-assessed research competency levels among operating room nurses after intervention [n (%), *n* = 103]

Dimension	Competent	Moderately Competent	Insufficiently Competent	Total
Thesis Writing Ability	49(47.6)	40(38.8)	14(13.6)	103
Research Design Competency	50(48.5)	41(39.8)	12(11.7)	103
Literature Retrieval Ability	79(76.7)	17(16.5)	7(6.8)	103
Problem Identification Ability	69(67.0)	28(27.2)	6(5.8)	103
Data Processing Ability	60(58.2)	38(36.9)	5(4.9)	103
Research Practice Ability	49(47.6)	50(48.5)	4(3.9)	103

**Table 6:** Comparison of scores on the research capacity self-assessment scale before and after intervention [M (P25, P75), *n* = 103]

Group	Thesis Writing Ability	Research Design Competency	Literature Retrieval Ability	Problem Identification Ability	Data Processing Ability	Research Practice Ability	Total Score
**Pre-intervention**	5(4,6)	4(3,7)	13(9,17)	8(6,9)	12(9,15)	13(11,17)	58(49,67)
**Post-intervention**	21(12,24)	20(13,20)	20(20,20)	12(10,12)	20(13,20)	21(12,24)	111(79,120)
*Z* value	-9.013	-9.898	-8.089	-8.848	-7.145	-5.413	-9.040
*P*-value	<0.001	<0.001	<0.001	<0.001	<0.001	<0.001	<0.001

### Comparison of research output

From 2022 to 2024, significant increases were observed in the number of approved research projects (from 2 to 10; *χ²* =6.100, *P* = 0.047), publications in core journals (from 4 to 14; *χ²* =6.383, *P* = 0.041), and publications in first-tier journals (from 0 to 6; Fisher’s exact test, *P* = 0.031). The number of patents granted showed no significant change, See Table 7.

**Table 7:** Comparison of research outputs among operating room nurses from 2022 to 2024 (*n* = 103)

Year	Research Projects Approved	Patents Granted	Papers in Core Journals	Papers in Tier 1Journals
2022	2(1.9)	4(3.9)	4(3.9)	0
2023	5(4.9)	6(5.8)	8(7.8)	2(1.9)
2024	10(9.7)	6(5.8)	14(13.6)	6(5.8)
Statistical test	*χ²* =6.100	*χ²* =0.527	*χ²*=6.383	Fisher’s exact test
*P*-value	0.047	0.768	0.041	0.031

### Comparison of intangible outcomes among QCC members

QCC members reported significantly higher self-assessment scores post-intervention across eight of nine intangible outcome dimensions (all *P* < 0.05), including learning initiative, problem-solving, and teamwork. The dimension “Broadening Knowledge Scope” showed a non-significant improvement (*P* = 0.146).

**Table 8:** Comparison of intangible outcome scores among QCC members before and after intervention [M (P25, P75), *n* = 9]

Dimension	Pre-intervention	Post-intervention	Z value	*P*-value
Learning Interest	3.0 (3.0, 3.0)	3.5 (3.0, 4.0)	2.031	0.042
Learning Initiative	4.0(3.5,4.5)	5.0(4.0,5.0)	2.041	0.041
Problem Identification & Solving Ability	4.0(3.0,4.0)	5.0(4.0,5.0)	2.584	0.010
Teamwork Ability	3.0(3.0,4.0)	4.0(4.0,5.0)	2.291	0.022
Broadening Knowledge Scope	4.0(3.0,4.5)	4.0(4.0,5.0)	1.455	0.146
Integrating Theory with Practice	4.0(3.0,4.0)	4.0(4.0,5.0)	2.062	0.039
Independent Thinking Ability	3.0(3.0,4.0)	5.0(4.0,5.0)	2.612	0.009
Language Expression Ability	3.0(3.0,4.0)	5.0(4.0,5.0)	2.244	0.025
Scientific Research Thinking Ability	4.0(3.0,4.0)	5.0(4.0,5.0)	2.318	0.020

**Fig. 6:**
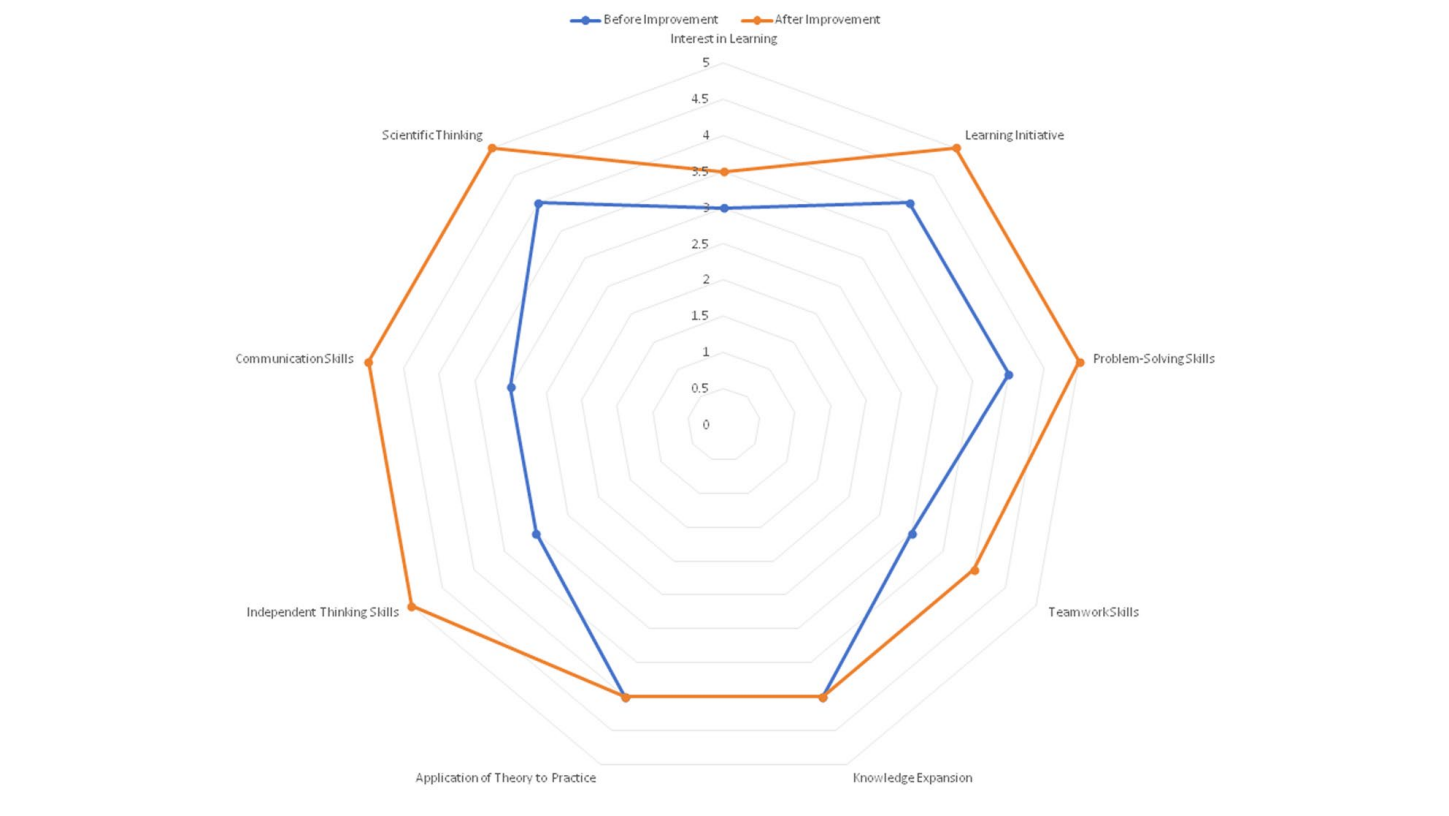
Radar chart comparing intangible outcomes before and after the intervention

To visually complement these statistical findings from Table 8, the changes in all intangible outcome dimensions are summarized in the radar chart presented in Fig. 6:

## Discussion

This study demonstrates that a hybrid training program integrating an enhanced PDSA cycle with QCC methodology can significantly improve both the perceived research competency and tangible scholarly output of operating room nurses. The intervention achieved high goal attainment (131.8%) and improvement (129.8%) rates, with benefits sustained over a two-year follow-up period.

### Expanding the PDSA application scope

While the PDSA cycle is widely used for standardizing clinical procedures [8–10, 17], this study innovatively applies it to developing complex “soft skills” like research capacity. Our findings confirm that its iterative “Plan-Do-Study-Act” logic is equally effective for systematic human capability development, extending its utility in nursing management beyond technical process optimization.

### A systematic framework for ‘soft skill’ cultivation

Unlike studies focusing on isolated training events [1–5], this intervention provided a structured, sustainable framework. By decomposing research capacity into measurable modules and managing it as a department-level QI project through QCC teams, we shifted the paradigm from dependent educational activities to empowered, data-driven system improvement. This integration of iterative action research principles with a formal QI structure represents a core methodological contribution.

### Contextually adapted program design

The program was tailored to the high-intensity, fragmented operating room context. Strategies like using the platform for micro-training, simulated workshops for theory-practice integration, and establishing a research committee directly addressed environmental barriers identified in prior surveys [4] and align with theories of situated learning [18]. This offers a replicable “contextualized solution template” for similar specialty settings.

### Building a sustainable research ecosystem

It is important to note that this sustained improvement in research outputs should be interpreted as an association with the intervention rather than a direct causal effect, as potential time‑related confounders (e.g., institutional research policy adjustments, career progression incentives) cannot be fully excluded in real‑world clinical settings.

### Strengths, limitations, and future directions

Self‑reported research competency scores may be subject to social desirability bias and the Hawthorne effect, as participants were aware of being part of a department‑wide quality improvement project led by senior staff, which may have led to potential inflation of post‑intervention assessment scores. Although objective research output data (accounting for 60% of the composite evaluation) were used for cross‑validation to mitigate such biases, the subjective nature of self‑assessment for intangible competencies cannot be completely eliminated.

The single‑center, pre‑post intervention design limits causal inference and generalizability; potential threats to internal validity include maturation effects (nurses’ natural professional growth), history effects (institutional research policy adjustments, increased academic incentives) and organizational changes during the study period, which may have contributed to the observed improvements in research capacity and output.

This study relies primarily on P‑values for statistical inference, without reporting effect sizes or 95% confidence intervals, which limits the interpretation of the magnitude and practical significance of the observed changes. Additionally, multiple outcome indicators were tested without formal correction for multiple comparisons, which may increase the risk of Type I error inflation.

Composite “Xiankuangzhi” index: This internally developed indicator is strictly an internal quality management tool for tracking our department’s QI intervention progress, with a weighting system (40% subjective, 60% objective) determined by QCC team consensus based on clinical QI practice principles in China. It lacks external validation and international recognition, and is not intended to be a standardized research outcome measure for cross‑study comparison.

The study was conducted in a single tertiary hospital in China with a homogeneous nursing workforce (all holding bachelor’s degrees) and a highly supportive organizational context (e.g., access to postgraduate mentors, digital collaboration platforms for training and mentoring). These contextual constraints limit the external applicability of the intervention model, which requires adaptation to local resources (e.g., staff educational background, research infrastructure) in other healthcare settings.

We will improve statistical rigor in future studies by incorporating effect sizes, 95% confidence intervals, and appropriate adjustments for multiple comparisons. We also plan to validate the “Xiankuangzhi” index using multicenter data and develop more objective research competency assessment tools to reduce biases associated with self‑reported data.

## Conclusion

A Quality Control Circle (QCC) intervention structured around an enhanced Plan‑Do‑Study‑Act (PDSA) cycle is a feasible strategy associated with improved research capacity, scholarly productivity, and associated competencies of operating room nurses in real‑world clinical settings, providing a practical, replicable framework for embedding a sustainable research culture within clinical nursing units, which requires appropriate adaptation based on local organizational and resource contexts.

## Supplementary Information

Below is the link to the electronic supplementary material.

Supplementary Material 1

## Data Availability

The datasets used and/or analyzed during the current study are available from the corresponding author on reasonable request.
