# Supplementary material for: Effectiveness of a Plan-Do-Study-Act cycle-based quality control circle in enhancing the research capacity of operating room nurses a pre-post intervention study
Source: BMC Nurs. 2026 Mar 25;25:417. doi: 10.1186/s12912-026-04566-3 (PMC13137706; doi:10.1186/s12912-026-04566-3)
Supplement: Supplementary file 1 — Supplementary Material 1 [file 12912_2026_4566_MOESM1_ESM.docx]

QCC Intervention: Detailed Operational Procedures

This section describes the detailed operational workflow of the Quality Control Circle (QCC) intervention based on an enhanced Plan‑Do‑Study‑Act (PDSA) cycle, which was simplified in the main manuscript.

1. Study Design & Participants

The QCC program was implemented as a single‑center, prospective, pre‑post intervention study among operating room nurses. All participants were registered nurses working full‑time in the operating room department, with similar professional backgrounds and clinical experience.

2. QCC Structure and Organization

A dedicated QCC team was established, consisting of:

- 1 QCC supervisor (senior nursing administrator)

​

- 1 QCC coordinator (head nurse)

​

- 4 QCC group leaders

​

- All participating operating room nurses as core members

3. Enhanced PDSA Cycle Implementation

Plan Stage

- Baseline assessment of research capacity, research outputs, and related competencies.

​

- Identification of weaknesses in research awareness, knowledge, skills, and clinical‑research integration.

​

- Development of a structured training and supervision plan.

Do Stage

- Regular group meetings (biweekly or monthly, adjusted according to departmental workflow).

​

- Targeted training on research design, literature retrieval, statistical analysis, and academic writing.

​

- One‑on‑one mentoring for research project initiation, data collection, and manuscript preparation.

​

- Construction of a digital platform for resource sharing, online communication, and progress tracking.

Study Stage

- Periodic evaluation of training effectiveness and participant progress.

​

- Comparative analysis of pre‑ and post‑intervention competency scores and research outputs.

​

- Summary of barriers encountered during implementation and corresponding solutions.

Act Stage

- Standardization of effective practices into routine departmental management.

​

- Development of a long‑term mechanism for sustainable research culture building.

​

- Adjustment and optimization of the intervention model for continuous quality improvement.

4. Scoring and Evaluation Criteria

The composite evaluation system included:

- 60% objective indicators: published papers, granted projects, patent applications, and other research outputs.

​

- 40% subjective indicators: self‑rated and peer‑rated research competency, learning attitude, and teamwork.

The internally developed “Xiankuangzhi” index was used only for internal quality improvement monitoring, not as a standardized tool for external comparison.

5. Quality Control

- Regular team meetings to ensure standardized implementation.

​

- Uniform training materials and assessment criteria.

​

- Cross‑checking of research output records to ensure authenticity.

​

- Continuous feedback and dynamic adjustment of the intervention.
